# Preparation of Curcumin-Eudragit^®^ E PO Solid Dispersions with Gradient Temperature through Hot-Melt Extrusion

**DOI:** 10.3390/molecules26164964

**Published:** 2021-08-17

**Authors:** Wenling Fan, Xiaotong Zhang, Wenjing Zhu, Xinyi Zhang, Liuqing Di

**Affiliations:** 1 Laboratory of Pharmacy Engineering, College of Pharmacy, Nanjing University of Chinese Medicine, Nanjing 210023, China; aixiaotong95@163.com (X.Z.); zwj19970114@163.com (W.Z.); 18260091268@163.com (X.Z.); 2 Jiangsu Collaborative Innovation Center of Chinese Medicinal Resources Industrialization, School of Pharmacy, Nanjing University of Chinese Medicine, Nanjing 210023, China; 3 Institute of Jiangsu Engineering Research Center for Efficient Delivery System of Traditional Chinese Medicine, School of Pharmacy, Nanjing University of Chinese Medicine, Nanjing 210023, China; diliuqing928@163.com

**Keywords:** insoluble, dissolution, thermo-sensitive

## Abstract

Hot-melt extrusion (HME) has great advantages for the preparation of solid dispersion (SD), for instance, it does not require any organic solvents. Nevertheless, its application to high-melting-point and thermosensitive drugs has been rarely reported. In this study, thermally unstable curcumin (Cur) was used as a drug model. The HME process was systematically studied by adjusting the gradient temperature mode and residence time, with the content, crystallinity and dissolution of Cur as the investigated factors. The effects of barrel temperature, screw speed and cooling rate on HME were also examined. Solubility parameters and the Flory–Huggins method were used to evaluate the miscibility between Cur and carriers. Differential scanning calorimetry, X-ray diffraction, Fourier transform infrared spectroscopy, equilibrium solubility and in vitro and in vivo experiments were used to characterize and evaluate the results. An amorphous Cur SD was successfully obtained, increasing the solubility and release of Cur. In the optimal process, the mass ratio of Cur to Eudragit^®^ E PO (EPO) was 1:4 and the barrel temperature was set at a gradient heating mode (130 °C–135 °C–140 °C–145 °C–150 °C–155 °C–160 °C) at 100 rpm. Related pharmacokinetic test results also showed the improved bioavailability of the drug in rats. In a pharmacodynamic analysis of Sprague–Dawley rats, the Cmax and the bioavailability of the Cur-EPO SD were 2.6 and 1.5 times higher than those of Cur, respectively. The preparation of the amorphous SD not only provided more solubility but also improved the bioavailability of Cur, which provides an effective way to improve the bioavailability of BCS II drugs.

## 1. Introduction

Curcumin (Cur) is a natural polyphenol, isolated from *Curcuma longa*, with a wide range of pharmacological effects, such as anti-inflammatory, anti-oxidant, anti-cancer and anti-microbial effects [1,2], and it has produced relatively low levels of adverse reactions during clinical applications [3,4]. Cur is a thermosensitive compound that degrades without reaching its melting point (Tm). However, its therapeutic efficacy is limited because of its poor solubility [5]. Several methods, such as the preparation of liposomes and nanoparticles [6,7] and self-microemulsion, have been used to increase the solubility and dissolution rate of insoluble drugs [8,9,10,11]. Solid dispersion (SD) can also efficiently increase the dissolution rate and solubility of insoluble drugs by changing the size and dispersion within the carrier. Compared with the abovementioned technologies, this strategy has advantages of high drug loading and suitability for industrial production [12,13]. To date, solvent evaporation has been applied to prepare thermosensitive Cur SDs [5,14,15,16]. Although good dissolution effects and bioavailability were obtained, issues such as solvent residues and industrial production must be considered. In the case of hot melt extrusion technology, there are no problems involved with the use of a large amount of solvents.

Hot-melt extrusion (HME) converts drugs and carriers into products at elevated temperatures and rapid shear rates to produce SDs and has been widely used in the pharmaceutical industry [17,18]. Compared with some conventional approaches, such as rotary evaporation, which can be costly and has several disadvantages, such as requiring additional organic solvents, raw materials and extra facilities for solvent removal and to mitigate the explosion risk. The remarkable industrial advantages of HME include the avoidance of organic solvents, the possibility of continuous manufacturing, the high uniformity of extrudates and the ease of processing and scaling-up [19]. However, thermosensitive drugs or carriers, which are degraded when overheated, are rarely used in HME. Adjusting the gradient temperature and reducing the residence time can effectively prevent the degradation of thermosensitive drugs and carriers to ensure product quality [20] In general, the zones of the barrel have the same temperature: a lower level may cause incomplete melting of the drug and poor dispersibility in the carrier, and a high temperature may degrade thermosensitive drugs or carriers. Only a few studies have prepared SDs of heat-sensitive drugs using HME. Mendonsa applied HME to mix poloxamer and ketoprofen and then developed a poloxamer gel. The temperatures of the barrel from zones 2–4 were set at 97 °C to ensure the melting of the drug and polymer, with zone 5 at 90 °C, zones 6 and 7 at 80 °C and zone 8 and the die set at 70 °C to ensure the correct form [21]. Chuah used HPMC, lecithin and isomalt as carriers to prepare an amorphous Cur SD through HME [22]. When the temperatures of different zones were set as 50 °C–100 °C–130 °C–140 °C–150 °C, no degradation occurred, and the obtained SD showed a good anti-inflammatory activity. However, a systematic study has not been conducted on temperature control when preparing SDs through HME.

Many thermosensetive and insoluble drugs, such as tanshinone IIA, vitamin C and Cur, have been developed. In this work, the HME process was systematically studied to explore its application for the preparation of SDs of heat-sensitive and insoluble drugs. Under gradient temperature control, a thermosensitive amorphous Cur SD was successfully prepared through HME without the degradation of the Cur. The SDs were characterized by means of differential scanning calorimetry (DSC), X-ray diffraction (XRD) and Fourier transform infrared spectroscopy (FTIR). Their stability and dissolution in vitro were also studied. The dissolution rate and bioavailability were significantly improved compared to the raw drug. Thus, this study can provide a useful reference for Cur research and the development of oral drug delivery systems. In this paper, an SD of the thermo-sensitive drug Cur was prepared using HME technology, expanding the scope of the application of HME technology and providing an example of the preparation of SDs by means of the HME of other thermo-sensitive drugs.

## 2. Materials and Methods

### 2.1. Materials

Cur with 95% purity was purchased from Shanghai Yuanye Biological Technology Co., Ltd. (Shanghai, China). Eudragit EPO (EPO), employed as an HME carrier, was provided by Evonik Industries AG (Darmstadt, Germany). Tween-80 and methanol were purchased from National Drug Group Chemical Reagents Co., Ltd. (Shanghai, China). Acetonitrile and phosphoric acid were supplied by EMD Millipore Corporation (Billerica, MA, USA). The acetonitrile and phosphoric acid were chromatographic grade. The other reagents were either of analytical grade.

Fifteen male Sprague–Dawley rats (180–220 g) were provided by the Experimental Animal Center of Nanjing University of Chinese Medicine (NJUCM, Certificate no. 201804676). The experimental procedures were in line with the requirements of the Animal Ethics Committee of the Nanjing University of Chinese Medicine.

### 2.2. Thermal Analysis

The thermal degradation temperatures of Cur and EPO were measured using a Pyris 1 thermogravimetric analyzer (PerKin Elmer, Waltham, MA, USA). Approximately 10 mg of the sample was placed in a small aluminum pan and heated from 30 °C to 250 °C at a heating rate of 10 °C/min. Nitrogen was used as a purge gas at a flow rate of 30 mL/min.

**Figure 1:**
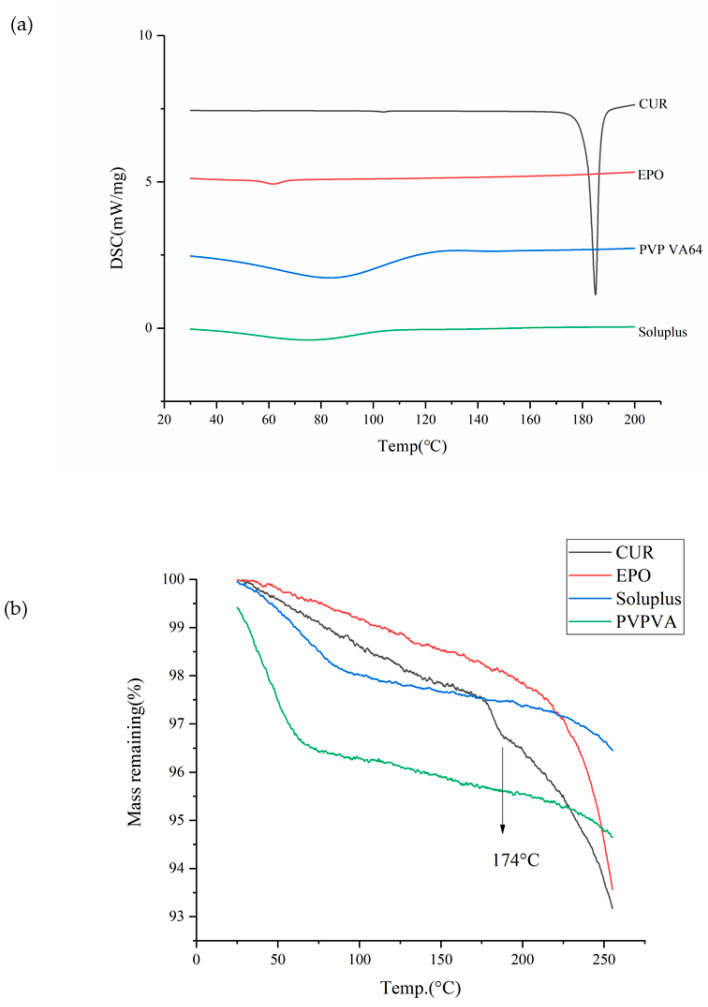
The DSC (**a**) and TGA (**b**) curves of samples.

Thermal behavior was examined using a DSC (NETZSCH 200 F3 thermo gravimetrical analyzer, NETZSCH group, Erlangen, Bayern, Germany). In brief, 5 mg of powder sample was packed into an aluminum pan with a pinhole in the lid to remove moisture and was heated at rate of 10 °C/min from 30 °C to 210 °C. Nitrogen at the flow rate of 30 mL/min was used as a purge gas. NETZSCH Proteus analysis software was used to analyze the data. The results are shown in Figure 1.

### 2.3. Miscibility Study of Drug and Carriers

#### Solubility Parameters

**Figure 2:**
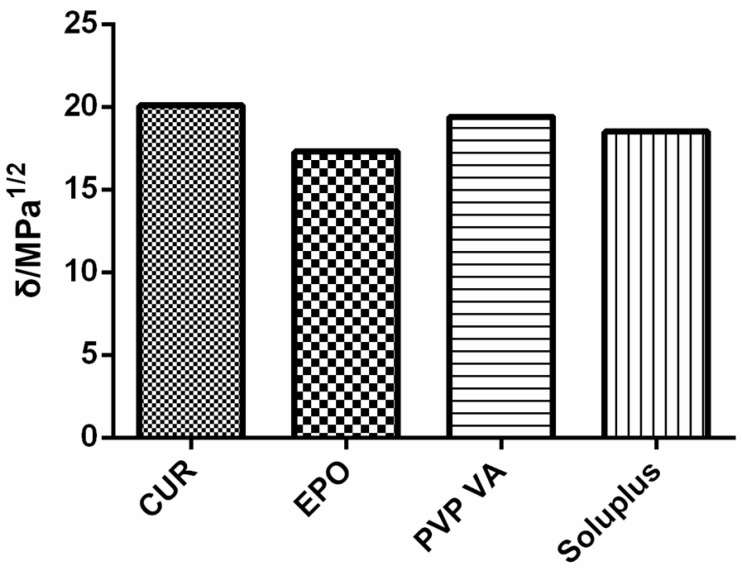
Solubility parameters of samples.

The solubility parameter δ, which was originally proposed by Hildebrand and Scott and which was improved and perfected by Small and Hansen [23], has been used to predict the miscibility of drugs and carriers since 1999 [24]. To date, the most frequently applied method uses Hansen–Hildebrand solubility parameters, which can be divided into dispersive interactions (δ_d_), polar interactions (δ_p_) and hydrogen bonding (δ_h_), and which are determined through a group-contribution method [25]. For Cur, the solubility parameters calculated on the basis of three different methods (Hoftyzer/Van Krevelen, Hoy, and Just) are shown in Figure 2.

(1) $$\delta =\sqrt{\delta_{d^{2}}+\delta_{p^{2}}+\delta_{h^{2}}}$$

### 2.4. The Preparation of the Cur-EPO System Using HME

#### 2.4.1. Preparation of Physical Mixture (PM)

The drug/carrier physical mixture was prepared with a mortar and pestle under liquid nitrogen. The powder was stored in a desiccator at room temperature for further studies.

#### 2.4.2. Preparation of Cur-EPO SD

Cur and EPO (1:4, *w*/*w*) was gently mixed with a mortar under liquid nitrogen. The mixture was slowly added in the hot-melt extruder (Thermo Scientific, Karlsruhe, Germany) with co-rotating 11 mm screws (length: 44 cm, L/D = 40) at a preset barrel temperature and screw speed. The extrudate was ground using a mortar under liquid nitrogen and then collected through 80 sieves in a desiccator for use. With the crystallinity, dissolution and content of the drug as evaluation indices, a single-factor test was used to select the appropriate barrel temperature, screw speed and cooling rate to optimize the preparation process and ensure the quality and stability of the SD.

### 2.5. Determination of Drug Content

Cur was quantified using the HPLC method (Waters Corporation, Milford, MA, USA). A Hedera ODS-2 C18 column (5 μm × 4.6 mm × 250 mm) was employed and maintained at 35 °C [26]. All measurements were performed with the injection volume of 10 μL and the UV wavelength set at 423 nm. Acetonitrile and 0.3% aqueous phosphoric acid solution (6:4, *v*/*v*) was used as the mobile phase and pumped at a flow rate of 1 mL/min. The calibration curve was linear over a range of 1.373–87.840 μg/mL, with a correlation coefficient of approximately 0.9998.

The drug or SD was sonicated with methanol and the supernatant was collected and analyzed through HPLC after centrifugation.

### 2.6. X-ray Powder Diffraction (XRD)

The samples were measured using a D/max 2500 X-ray powder diffraction system (Rigaku, Tokyo, Japan) to determine the molecular transformation of the drug from the crystalline to the amorphous state. Cu Kα radiation was used at 40 kV and 100 mA. The samples were scanned in the reflection mode from 3° to 40° using a scanning step size of 0.02°.

### 2.7. Fourier Transform Infrared Spectroscopy (FTIR)

A NEXUS870 FTIR (Thermo Nicolet, Madison, WI, USA) instrument was used to confirm the existence of molecular interactions between the drug and polymer. The samples were gently mixed with dry potassium bromide, compressed and analyzed from 4000 cm^−1^ to 400 cm^−1^. Each disc was scanned 10 times at a resolution of 2 cm^−1^.

### 2.8. In Vitro Dissolution Study

In vitro dissolution was conducted using a ZRS-8 GD paddle dissolution apparatus (Tiandatianfa, Tianjin, China) with a paddle rotation speed of 100 rpm at 37 °C ± 0.5 °C in 900 mL of pH 1.2 HCl solution according to the *Chinese Pharmacopoeia*, 2020, English version. Samples equivalent to 12 mg of Cur were added to the dissolution apparatus. Afterwards, 3 mL of samples were withdrawn at predetermined time intervals, and an equal amount of fresh medium was added to the dissolution medium vessel. The collected samples were centrifuged at 14,000 rpm for 10 min, and the supernatant was determined by means of HPLC with a detection wavelength of 306 nm. Experiments were performed in triplicate.

### 2.9. Equilibrium Solubility Study

An excess amount of crystalline Cur was dispersed in the pH 1.2 HCl solution with the presence and absence of EPO at 37 °C. Afterward, 5 mL of samples were withdrawn from each vessel after 24 h and centrifuged 14,000 rpm (Vortex Kylin-bell5 Vortex oscillator, Beideng group, China) for 10 min. The supernatant was determined by means of HPLC with a detection wavelength of 432 nm. Experiments were conducted in triplicate.

### 2.10. Accelerate Stability Study

The extrudates were stored in a Climacell 222 humidity chamber (MMM group, Planegg, Germany) at 40 °C and 75% relative humidity (RH) for 6 months to investigate their physical stability. DSC studies were utilized to regularly determine the crystallinity of Cur.

### 2.11. In Vivo Pharmacokinetic Studies

Fifteen rats were randomly divided into three groups, which were orally administered with 100 mg/kg pure Cur, the physical mixture of Cur-EPO, and the Cur-EPO SD [27,28]. In brief, 0.5 mL of blood samples were obtained from the orbital veins using a heparinized tube at different times of 5, 10, 15, 20, 30, 45, 60, 90, 120, 240, 360, 480, 600 and 1440 min and then centrifuged immediately at 4500 rpm for 10 min [29]. The plasma samples were collected and stored at −20 °C for further experiments. Afterwards, 10 μL of apigenin solution (internal standard, 0.403 μg/mL), 10 μL of ethyl acetate, and 100 μL of plasma sample were added into a 1.5 mL Eppendorf (EP) tube, vigorously vortexed and mixed for 3 min and centrifuged at 14,000 r/min for 10 min. Approximately 80 μL of supernatant was taken for the UPLC-MS/MS analysis. The DAS 3.0 pharmacokinetic program was used to analyze the pharmacokinetic parameters.

## 3. Results and Discussion

### 3.1. Thermal Analysis

Knowledge of the the thermodynamic properties of drugs is essential for the preparation of SDs through HME [30]. As shown in Figure 1a, the endothermic peak of Cur occurred at 181.5 °C (T_m_); however, significant thermal degradation is shown at 174 °C (T_d_) in Figure 1b without reaching the T_m_, thus verifying Cur as a thermosensitive drug. The glass transition temperature values for EPO, PVP VA and soluplus were 56.1 °C, 106 °C and 75.6 °C. The TGA thermogram of PVP VA showed a weight loss of approximately 4% between 50 °C and 100 °C; this may due to the evaporation of water, since PVP VA is highly hygroscopic. According to the thermodynamic properties of Cur, an appropriate barrel temperature should be selected to ensure that the drug–carrier system melts, and this was set below 174 °C to avoid the degradation of Cur.

### 3.2. Miscibility Study

- *Solubility Parameters*

According to the theory proposed by Greenhalgh [31], the miscibility of the drug and carrier depends on the difference in their Δδt values. Compounds with similar values of δ are miscible because the energy of mixing released by interactions within the components is balanced by the energy released by the interaction between the components [32]. Figure 2 shows that all calculated Δδt values were <7. With regard to the total solubility parameter, the difference in Δδt between Cur and the carrier was small, thus denoting miscibility.

### 3.3. Preparation of Cur-EPO SD

The weight ratio of Cur to EPO was 1:4. The HME process is important because Cur is easily decomposed at high temperatures.

#### 3.3.1. The Determination of the Carrier and the Ratio of the Carrier and the Drug

**Figure 3:**
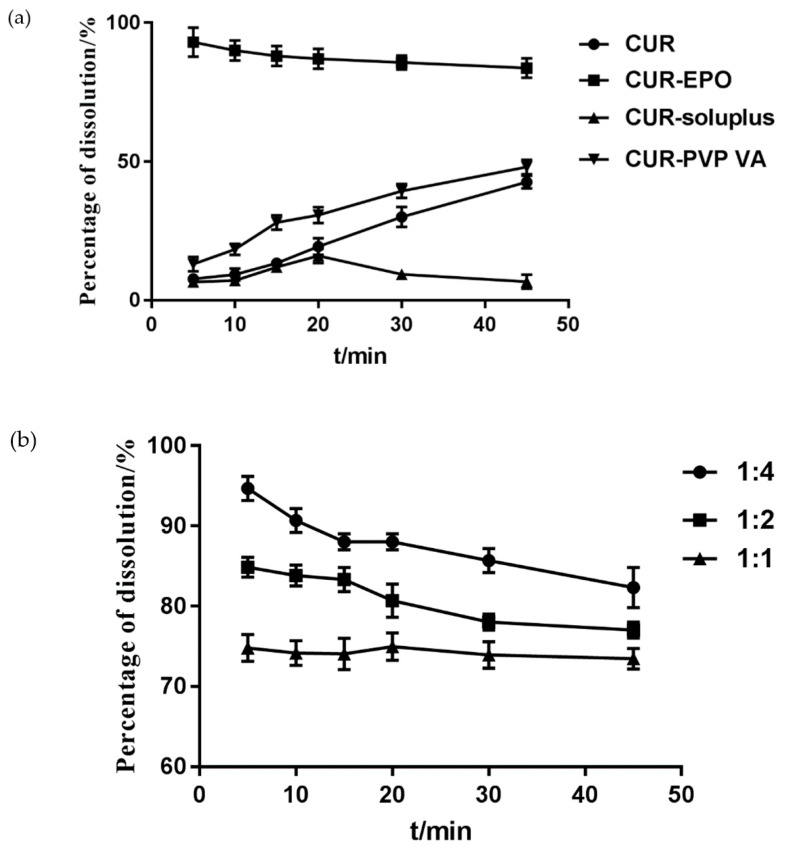
The determination of the carrier (**a**) and the ratio of the carrier (**b**).

The effects of carriers were investigated with the cumulative dissolution rate of Cur as the evaluation index. As shown in Figure 3, the incorporation of EPO into the Cur-SD substantially improved the Cur dissolution rate. Therefore, EPO was selected as the carrier of the Cur SD, and the ratio of the Cur-EPO was 1:4.

#### 3.3.2. Single Factor Test for Preparation Process

- *Effect of Barrel Temperature*

**Figure 4:**
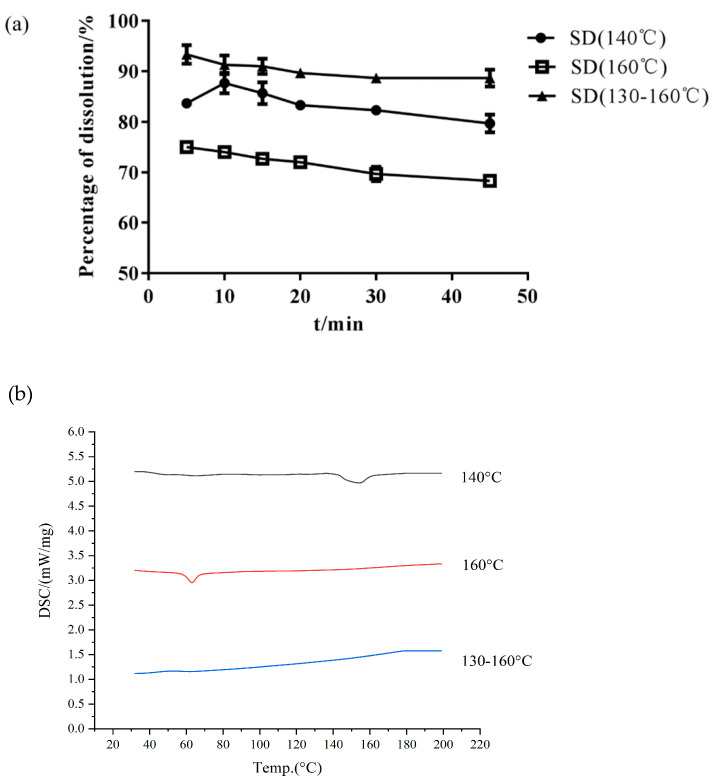
The dissolution of SD (**a**) and DSC curves (**b**) under different temperature control conditions.

Choosing the proper barrel temperature is important in preparing amorphous SDs and improving Cur dissolution [33]. HME may produce a residue of the crystalline drug at a low extrusion temperature or degrade the drug at a high temperature. Isothermal (140 °C and 160 °C) and gradient (130 °C–135 °C–140 °C–145 °C–150 °C–155 °C–160 °C) temperature methods were used to prepare the Cur-EPO SD through HME. As shown in Table 1 and Figure 4, the preparation of different temperature control methods greatly affected the thermal stability of the drugs. The content of raw Cur was 95%. When the barrel temperature was set to 160 °C, the drug was degraded, and the content of Cur was only 80%. Cur almost had no degradation when the barrel temperature was 140 °C and a temperature gradient (130 °C–135 °C–140 °C–145 °C–150 °C–155 °C–160 °C) was used. However, the related DSC curves (Figure 4) showed that when the barrel temperature was 140 °C, crystals still existed in the SD, and the corresponding cumulative dissolution rate was low. Therefore, the barrel temperature was set as a gradient heating mode (130 °C–135 °C–140 °C–145 °C–150 °C–155 °C–160 °C).

- *Effect of Screw Speed*

**Figure 5:**
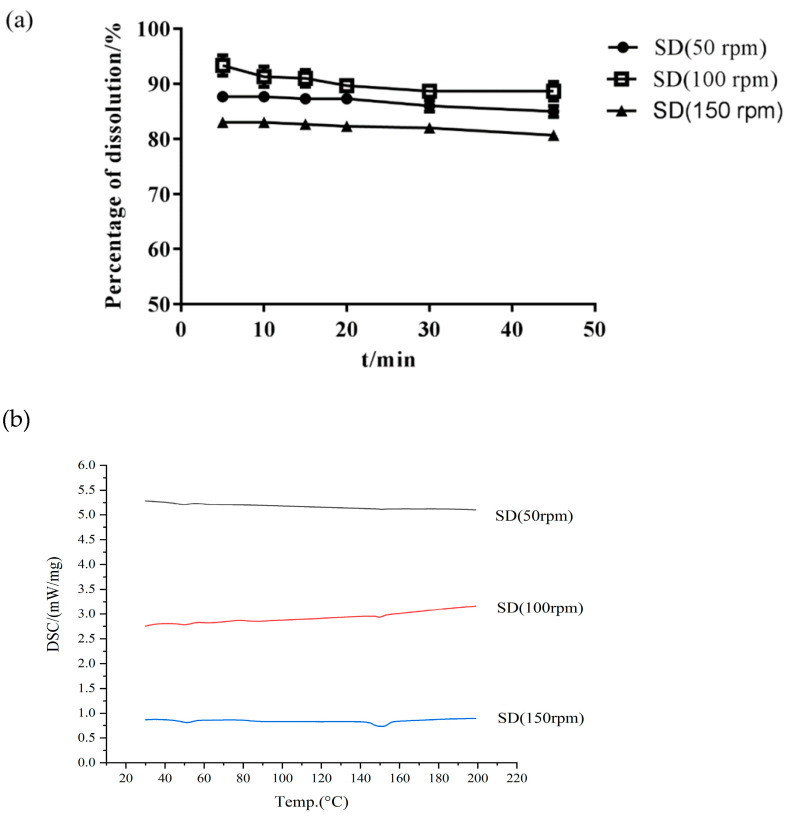
The dissolution of SD (**a**) and DSC curves (**b**) at different screw speeds.

The screw speed is another factor affecting the final product and its stability [34]. Table 1 and Figure 5 show that the majority of Cur degradation occurred at the screw speed of 50 rpm, possibly due to the low screw speed. When the speed was extremely low, the drug stayed in the barrel for a long time and then became extremely prone to thermal degradation. Figure 5 shows that when the barrel speed was 150 rpm, a crystal peak still occurred in the SD. An extremely fast screw speed (150 rpm) was not able to obtain the desired Cur-EPO SD and caused a short residence time of Cur in the barrel; the thermal energy and shear force provided by the instrument were unable to destroy the crystal lattice of the drug [35] When the screw speed was 100 rpm, Cur existed in an amorphous form, and its dissolution reached the highest rate (Figure 5).

- *Effect of Cooling Method*

**Figure 6:**
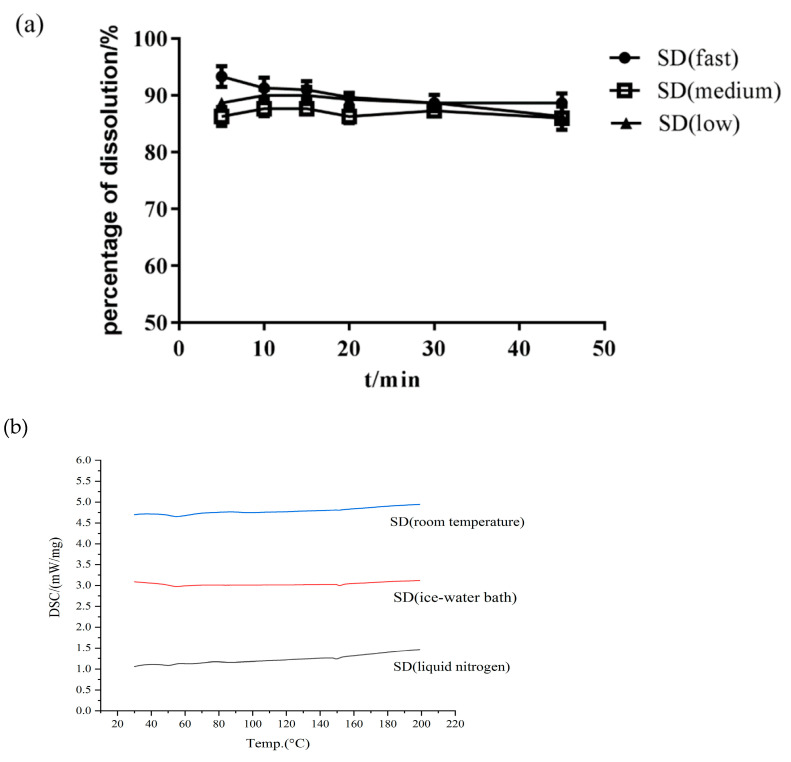
The dissolution of SD (**a**) and DSC curves (**b**) with different cooling methods.

The cooling method is also an important process involved in HME. An appropriate cooling method can prevent potential phase separation and drug nucleation and can thus be used to obtain amorphous SDs [36]. When the barrel temperature and screw speed were constant, the effect of the cooling method on the Cur-EPO SD was not remarkable (Figure 6). The mass fraction of Cur in all three cooling methods was approximately 95%, and almost no thermal degradation occurred. No significant difference in dissolution was observed in the presence of the carrier. Therefore, the final choice of cooling method was liquid nitrogen cooling.

The best preparation process was as follows: a screw speed of 100 rpm, a gradient temperature of 130 °C–135 °C–140 °C–145 °C–150 °C–155 °C–160 °C, and cooling by liquid nitrogen. Under such conditions, the drug was dispersed in the carrier in an amorphous state, and a strong molecular interaction occurred between the drugs and carriers.

### 3.4. The Characterization of the SD

#### 3.4.1. DSC

**Figure 7:**
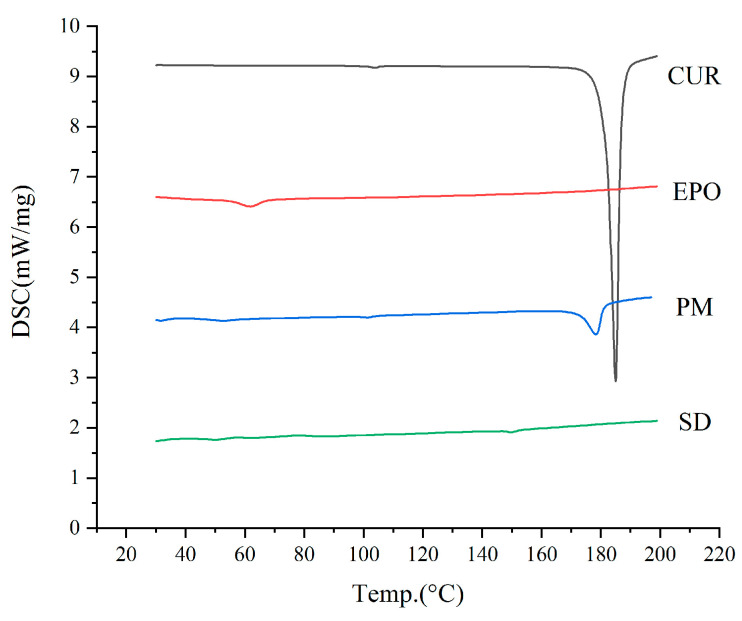
The DSC curves of the samples.

DSC was carried out to assess the crystalline state of raw Cur powder, EPO, the Cur and EPO physical mixture, and the Cur-EPO samples. As is shown in Figure 7, a sharp melting endothermic peak appeared at 181.8 °C in the DSC curve of raw Cur, which stemmed from the melting of Cur crystals. The DSC thermogram of the physical mixture showed a shallow endothermic peak at 171.5 °C, indicating the presence of Cur crystals. The melting peaks of Cur were broadened and shifted more to a lower temperature, which could be attributable to the mixing effect [37]. By contrast, the SD of the Cur-EPO samples presented no endothermic peak around the melting point of the crystals. It should be noted that when the crystallinity of a drug is under 2%, the melting peaks of the drug cannot generally be detected via DSC [38]. For this reason, further studies using XRD were carried out to assess the crystallinity of the SD.

#### 3.4.2. XRD

**Figure 8:**
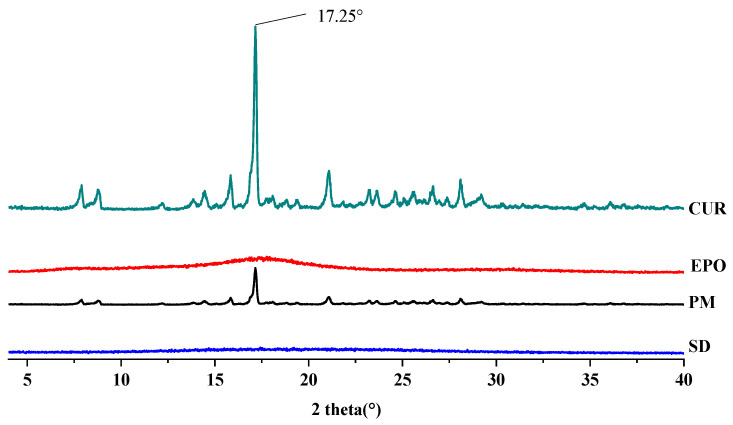
The XRD diffraction patterns of samples.

The XRD patterns of the samples are depicted in Figure 8. Cur showed the characteristic crystalline peaks of 2θ = 8.80°, 14.46°, 17.24°, 17.78°, 18.00°, 21.06°, 23.30°, 24.58°, 27.38° and 29.20° [39]. The X-ray diffractogram of EPO is typical of amorphous materials, with no sharp peaks [40]. There was a very strong peak at 17.25° 2θ in the curve of Cur. Corresponding signals also appeared in the physical mixture (PM) at the same angle, although these were weakened. On the contrary, no sharp crystal diffraction peaks were found in the SD, which means that amorphous Cur-EPO SDs were successfully prepared. A similar phenomenon has been reported by Li [41].

#### 3.4.3. FTIR

**Figure 9:**
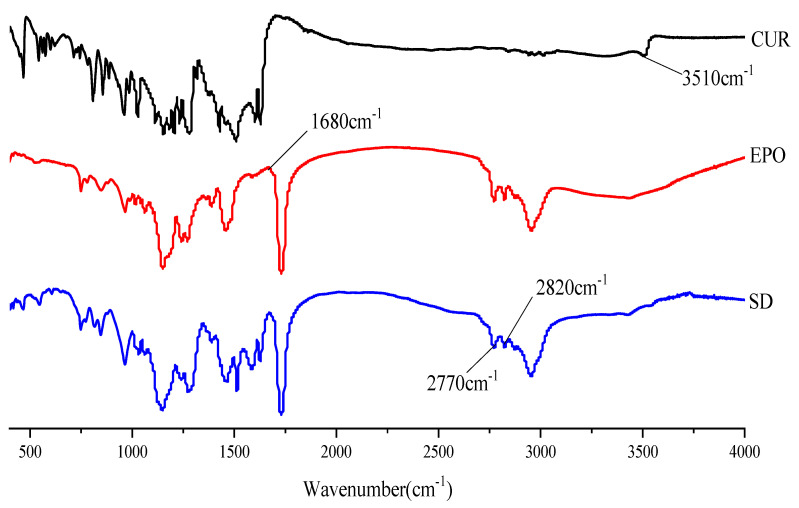
The FTTR spectrum of samples.

FTIR studies were conducted to confirm the presence of interactions between Cur and EPO. As can be seen in Figure 9, there was a broad peak at 3510 cm^−1^, which was assigned to phenolic hydroxyl group stretching of Cur [42]. The FTIR-spectra of pure EPO show the presence of two characteristic bands at 2770 and 2820 cm^−1^, corresponding to valence vibrations of non-ionized dimethylamino groups [43]. An absorption peak at 1680 cm^−1^ was assigned to the carbonyl group of EPO. In the spectrum of the SD, the bands at 3510 cm^−1^ disappeared and the carbonyl peak of EPO was shifted to lower wavelengths, which indicates that a hydrogen bond interaction between Cur and EPO may have occurred [44,45]. In addition, the stretching vibration peak intensity of EPO in the SD at 2770 cm^−1^ and 2820 cm^−1^ was also weakened. This might be due to the interaction of the protonated dimethylamino group from EPO with the phenolic hydroxyl group from Cur, which is similar to those published in the literature [46].

### 3.5. In Vitro Dissolution Study

**Figure 10:**
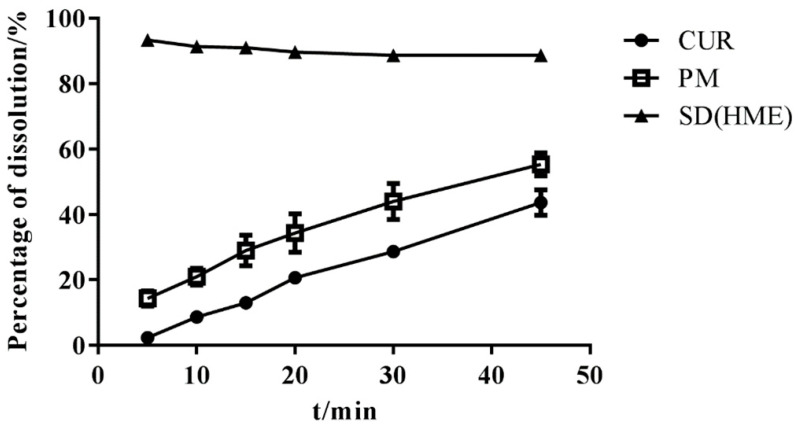
The dissolution of samples (n = 3).

The content of three batches of SDs was almost 95%, as detected using HPLC, and SDs were subjected to a dissolution test. As shown in Figure 10, the SD was completely released within 1 h and no degradation of Cur occurred, which means that immediate-release Cur-EPO SDs with no degradation were successfully prepared via HME.

### 3.6. Equilibrium Solubility Studies

Cur solubility was extremely poor (HPLC results could not be effectively measured); hence, 0.5% Tween was added to the dissolution medium. Excess Cur raw drug, the physical mixture, and the SD were added to a hydrochloric acid solution with pH 1.2, containing 0.5% Tween-80, and the equilibrium solubility is shown in Table 2. The solubility of the Cur-EPO SD was five times higher than that of Cur, indicating that the SD preparation can effectively improve Cur solubility [47,48].

### 3.7. Acceleration of Stability Study

**Figure 11:**
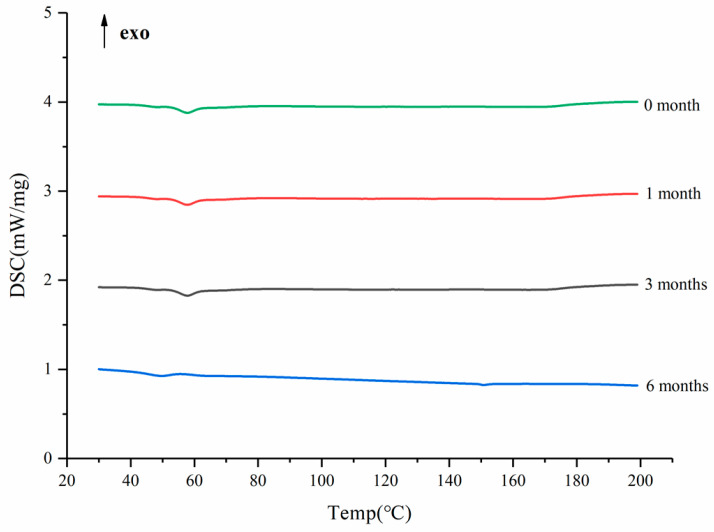
The DSC curves of the SD in different months.

After the SDs were subjected to the environment of 40 °C and RH75% for a period of time, the stability of the SDs were determined using DSC, and the results are shown in Figure 11. The DSC curves confirmed no recrystallization of the amorphous drug in the SDs, suggesting good physical stability [49]. This phenomenon could be ascribed to the molecular interaction and good miscibility between the drug and carrier.

### 3.8. Pharmacokinetic Study

**Figure 12:**
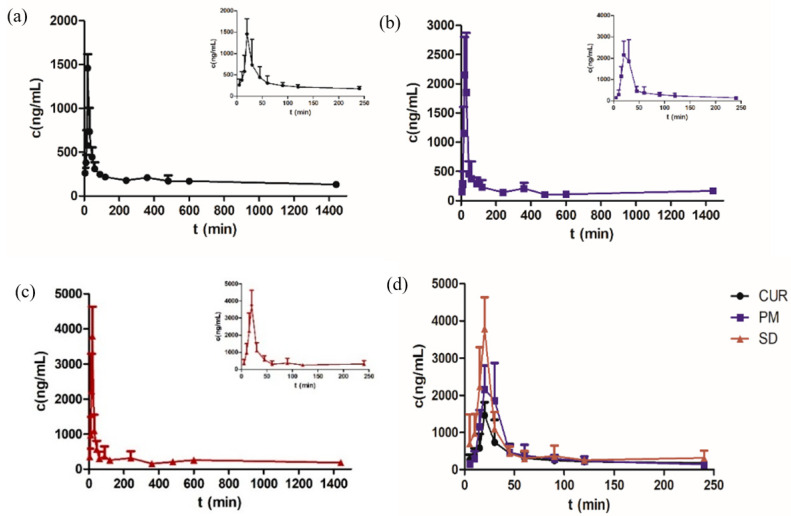
The plasma concentration-time curves of Cur in rats after oral administration of Cur (**a**), PM (**b**), the SD (**c**) and Cur, PM, SD (**d**) (n = 5, mean ± SD).

**Figure 13:**
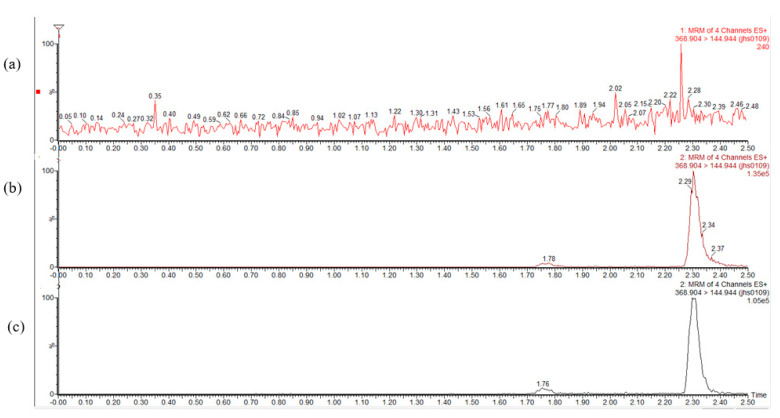
Mass spectrograms of Cur in blank plasma (**a**), blank plasma added reference (**b**) and plasma after administration (**c**).

The mean plasma concentrations are shown in Figure 12 and Table 3. The C_max_ of the SD was 3791.120 ± 22.2 ng/mL, which was 2.6 times higher than that of Cur. No significant difference was found between the physical mixture (PM) and Cur. The bioavailability of the Cur-EPO SD was 1.5 times higher than that of Cur and therefore was consistent with the solubility and dissolution results. Pharmacokinetics indicated that the preparation of Cur-EPO SD via HME can enhance the bioavailability to some degree due to its rapid metabolism in the liver and intestines [50,51]. Additional works must be conducted to increase the bioavailability of the prepared SDs. It can be seen in Figure 13 that endogenous substances in plasma show no interference with Cur, and the retention time is consistent with that of the control, indicating that the specificity of this method is good.

## 4. Discussion

Cur has a poor thermal stability, which makes it difficult to prepare a Cur SD through HME. The thermal decomposition of drugs mainly depends on the heating temperature and exposure time. Hot-melt extruders have an independent temperature control cylinder, which can set different temperatures in order to control the degree of heating of the drug, and a twin-screw structure, which can be used to control the heating time of the drug by setting the screw speed. The barrel temperature and screw speed must be adjusted to control the heating rate and barrel time of the drug [52]. The ability to prepare a thermosensitive drug as an SD by means of HME depends on two key factors, namely, barrel temperature and screw speed. In this experiment, a gradient temperature control mode was used to set the barrel temperature, adjust the appropriate screw speed, control the degree and time of heating, and reduce the probability of thermal degradation of the drug. The thermal properties of Cur were changed in the SD, namely, the single endothermic peak disappeared, and Cur was distributed in the carrier in an amorphous state, thus increasing the solubility and release of Cur [53]. Related pharmacokinetic test results also showed the improved bioavailability of the drug in rats.

## 5. Conclusions

In summary, under the gradient temperature control, an amorphous Cur-EPO SD with no degradation was successfully prepared through HME. After formulation as ASDs, the in vitro dissolution of the drug was remarkably increased, as compared to the bulk drug. The dissolution rate and bioavailability were significantly improved compared to the raw drug, which can provide a useful reference for Cur research and for the development of oral drug delivery systems. Physicochemical characterization by means of DSC and XRD analysis revealed that Cur was in an amorphous state and that all the ingredients in the formulation were compatible. Related pharmacokinetic test results showed that SDs could achieve better bioavailability of poorly-water-soluble drugs (BSC class II drugs). Nevertheless, the question of whether these design strategies can indeed provide better oral bioavailability still needs to be confirmed through preclinical and clinical studies.

## Figures and Tables

**Table 1:** The preparation process of the Cur-EPO SD.

	Barrel Temperature/°C	Screw Speed/rmp	Cooling Method	The Content of Cur/%
1	140	100	Room temperature	95
2	130–135–140–145–150–155–160	100	Room temperature	95
3	160	100	Room temperature	80
4	130–135–140–145–150–155–160	50	Room temperature	92
5	130–135–140–145–150–155–160	150	Room temperature	94
6	130–135–140–145–150–155–160	100	Ice-water bath	95
7	130–135–140–145–150–155–160	100	liquid nitrogen	95

**Table 2:** The equilibrium solubility of Cur (mean ± RSD, n = 3).

Samples	Equilibrium Solubility of Cur (μg/mL)
Cur	39.03% ± 0.63%
Physical mixture	152.94% ± 1.45%
SD	234.20% ± 2.33%

**Table 3:** The pharmacokinetic parameters of raw Cur, PM and the SD in rats after oral administration (n = 5, mean ± RSD).

Parameters	Cur	PM *	SD **
C_max_ (ng/mL)	1464.322 ± 24.7	2484.783 ± 26.8	3791.120 ± 22.2
T_max_ (min)	22 ± 22.3	24 ± 22.8	20 ± 0
AUC_0–t_ (ng/mL min)	267,081.009 ± 32.5	265,975.190 ± 21.5	387,231.473 ± 17.2
AUC_0–∞_ (ng/mL min)	533,813.494 ± 67.0	275,084.394 ± 15.8	619,877.046 ± 39.7
*t_1/2 z_* (h)	23.00 ± 53.8	7.03 ± 91.9	12.82 ± 35.7

Compare to raw Cur, * *p* > 0.05, ** *p* < 0.01.

## Data Availability

The raw data is available from the authors upon request.
